# Supplementary material for: Temporal and Site-Specific ADP-Ribosylation Dynamics upon Different Genotoxic Stresses
Source: Cells. 2021 Oct 28;10(11):2927. doi: 10.3390/cells10112927 (PMC8616546; doi:10.3390/cells10112927)
Supplement: Supplementary file 1 [file cells-10-02927-s001.zip › Buch-Larsen_suppl..pdf]

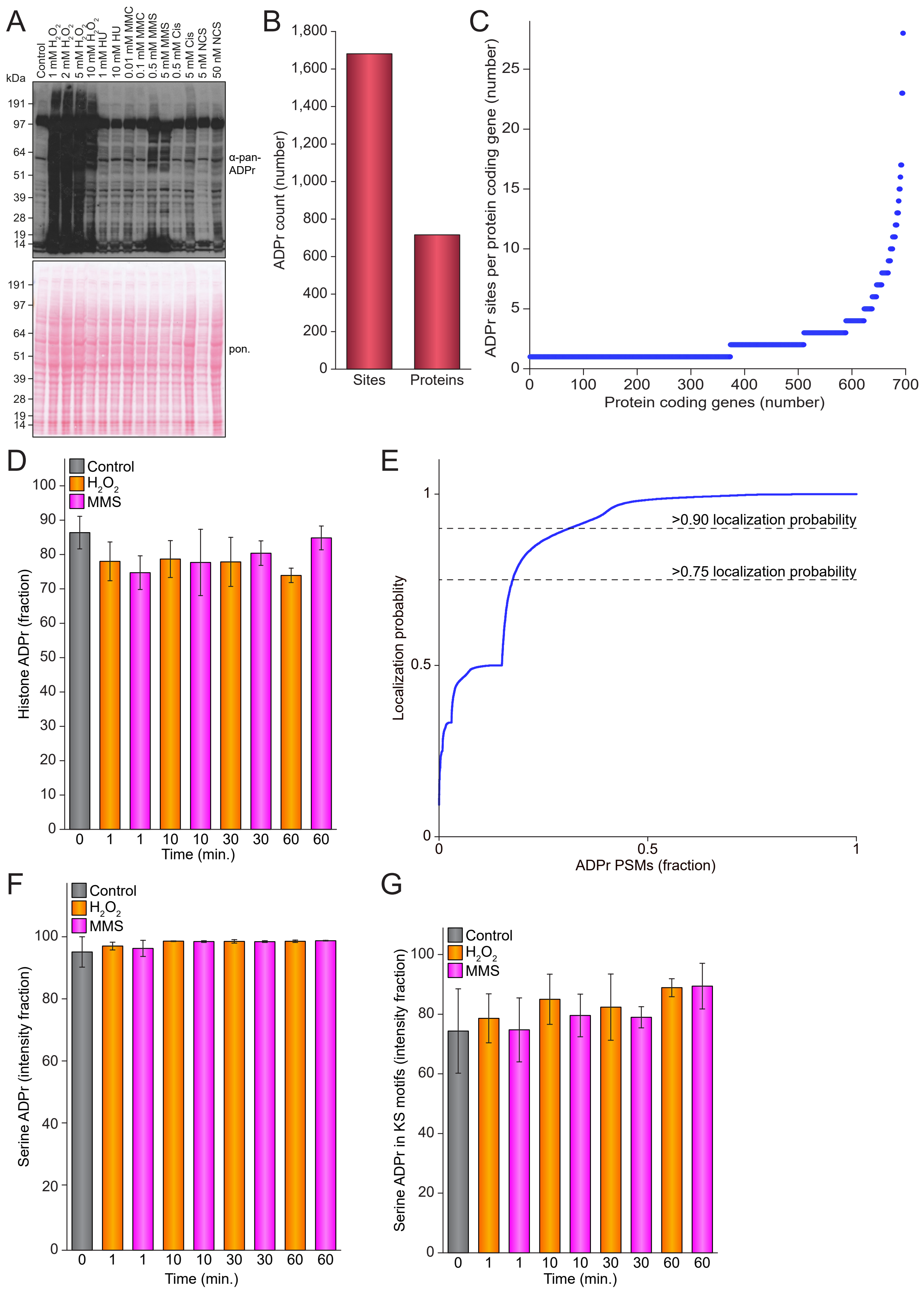

A
Control
1 mM H2O2
2 mM H2O2
5 mM H2O2
10 mM H2O2
1 mM HU
10 mM HU
0.01 mM MMC
0.1 mM MMC
0.5 mM MMS
5 mM MMS
0.5 mM Cis
5 mM Cis
5 nM NCS
50 nM NCS
kDa
191
97
64
51
39
28
19
14
α-pan-ADPr
pon.
B
ADPr count (number)
Sites
Proteins
C
ADPr sites per protein coding gene (number)
Protein coding genes (number)
D
Control
H2O2
MMS
Histone ADPr (fraction)
Time (min.)
E
Localization probability
>0.90 localization probability
>0.75 localization probability
ADPr PSMs (fraction)
F
Serine ADPr (intensity fraction)
Time (min.)
G
Serine ADPr in KS motifs (intensity fraction)
Time (min.)


FIGURE S1

**Figure S1. Induction of the ADPr signaling response.** A) Immunoblot analysis illustrating the ADPr equilibrium upon treatment with various concentrations of hydrogen peroxide ($H_2O_2$), hydroxyurea (HU), mitomycin C (MMC), methyl methanesulfonate (MMS), cisplatin (Cis), and neocarzinostatin (NCS) in HeLa cells. B) Histogram showing the total number of ADPr sites and proteins identified. C) Overview of the number of ADP-ribosylation sites per protein coding gene, plotted against the total number of identified protein coding genes ranked by site number. D) Overview of the fraction of ADPr residing on histones. *n*=4 cell culture replicates, data is presented as mean values +/− SD. E) ADPr localization probability plotted against the ranked fraction of all peptide-spectrum-matches (PSMs). Note, only PSMs with >0.9 localization probability were used to assign ADPr sites, whereas PSMs with >0.75 localization probability were additionally used to assign ADPr intensity. F) As **D**, but showing the fraction of ADPr residing on serine residues instead. G) As **F**, but illustrating the fraction of serine ADPr residing in KS motifs.

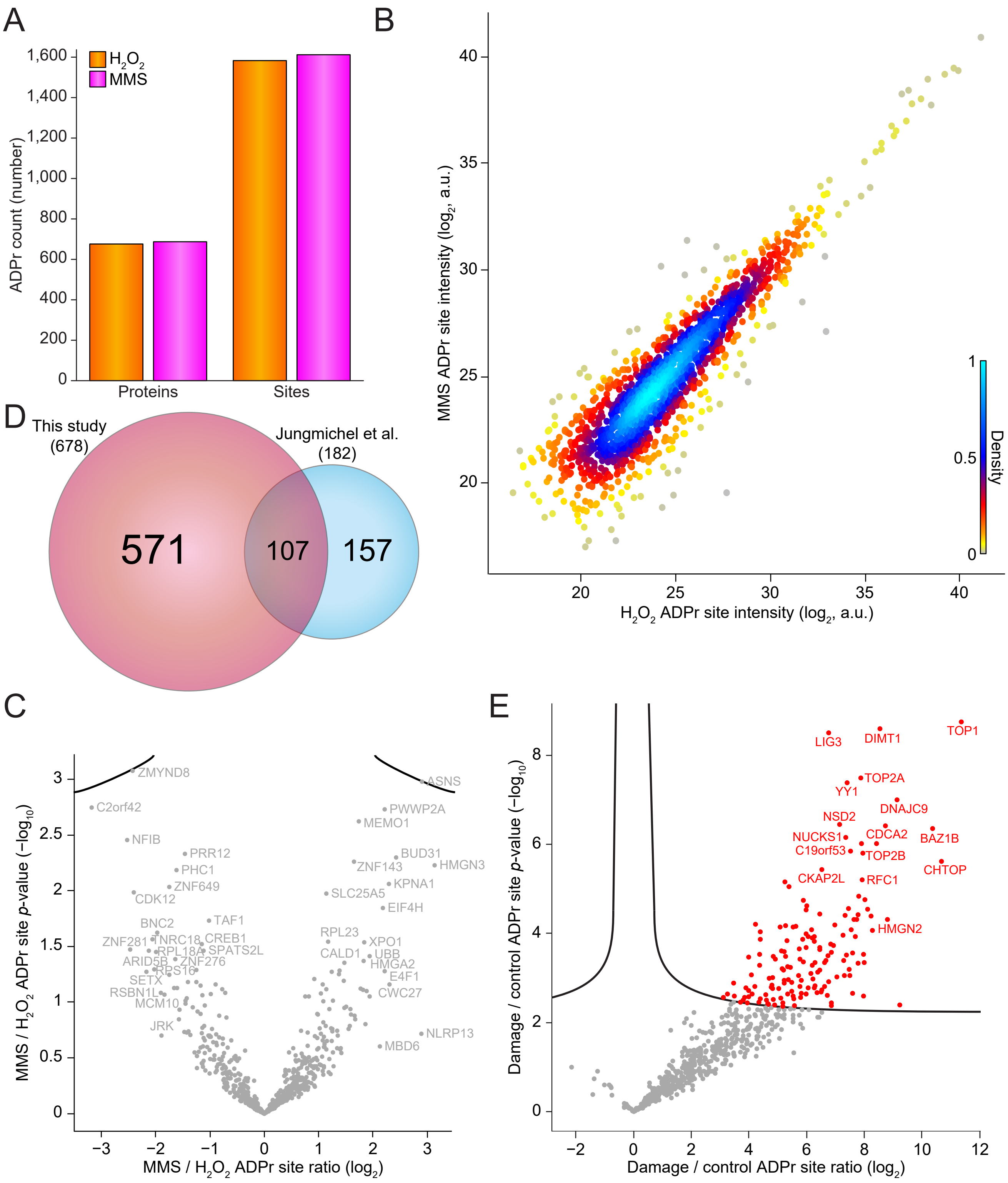
A
1,600
1,400
1,200
1,000
800
600
400
200
0
ADPr count (number)
H2O2
MMS
Proteins
Sites
B
40
35
30
25
20
MMS ADPr site intensity (log2, a.u.)
H2O2 ADPr site intensity (log2, a.u.)
1
0.5
0
Density
D
This study
(678)
Jungmichel et al.
(182)
571
107
157
C
MMS / H2O2 ADPr site p-value (−log10)
MMS / H2O2 ADPr site ratio (log2)
ZMYND8
C2orf42
NFIB
PRR12
PHC1
ZNF649
CDK12
BNC2
TAF1
ZNF281
TNRC18
CREB1
RPL18A
SPATS2L
ARID5B
ZNF276
RPS16
SETX
RSBN1L
MCM10
JRK
ASNS
PWWP2A
MEMO1
BUD31
ZNF143
HMGN3
KPNA1
SLC25A5
EIF4H
RPL23
XPO1
CALD1
UBB
HMGA2
E4F1
CWC27
NLRP13
MBD6
E
Damage / control ADPr site p-value (−log10)
Damage / control ADPr site ratio (log2)
LIG3
DIMT1
TOP1
TOP2A
YY1
DNAJC9
NSD2
NUCKS1
CDCA2
BAZ1B
C19orf53
TOP2B
CHTOP
CKAP2L
RFC1
HMGN2

FIGURE S2

**Figure S2. Damage-specific properties of the ADP-ribosylome.** A) Histogram illustrating the total number of ADPr sites and proteins identified upon $H_2O_2$ and MMS, respectively. B) Density scatter plot illustrating the correlation of ADPr sites identified under $H_2O_2$ and MMS treatment. C) Volcano plot analysis visualizing the dynamics of ADPr target proteins after $H_2O_2$ treatment compared to MMS treatment. Significance was determined via two-tailed Student's t-testing, with a FDR of 0.05, an s0 of 0.1, and 2,500 rounds of randomization. D) Scaled Venn diagram showing the overlap of ADPr-modified proteins identified in this study and the study by Jungmichel (Jungmichel et al., 2013). E) As **C**, but illustrating the dynamics after $H_2O_2$ or MMS treatment compared to control conditions. Proteins that are significantly upregulated upon genotoxic stress are depicted in red.

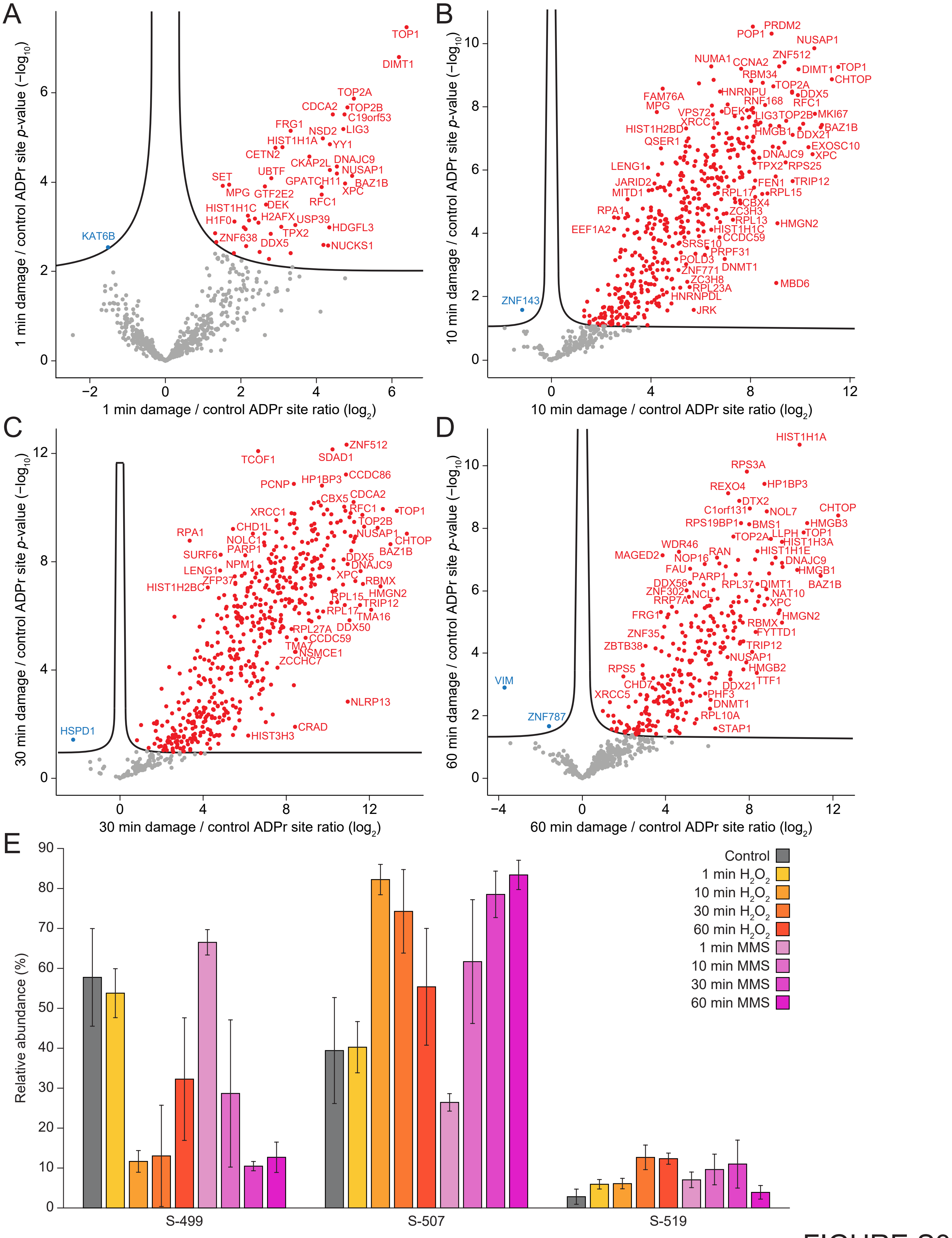
A
1 min damage / control ADPr site p-value (−log10)
1 min damage / control ADPr site ratio (log2)
B
10 min damage / control ADPr site p-value (−log10)
10 min damage / control ADPr site ratio (log2)
C
30 min damage / control ADPr site p-value (−log10)
30 min damage / control ADPr site ratio (log2)
D
60 min damage / control ADPr site p-value (−log10)
60 min damage / control ADPr site ratio (log2)
E
Relative abundance (%)
Control
1 min H2O2
10 min H2O2
30 min H2O2
60 min H2O2
1 min MMS
10 min MMS
30 min MMS
60 min MMS
S-499
S-507
S-519

FIGURE S3

**Figure S3. General temporal properties of the $H_2O_2$- and MMS-induced ADP-ribosylome.** A) Volcano plot analysis visualizing the dynamics of ADPr target proteins after 1 min of damage compared to control conditions. Significance was determined via two-tailed Student's t-testing, with a FDR of 0.05, an s0 of 0.1, and 2,500 rounds of randomization. Proteins significantly upregulated upon damage are depicted in red, and proteins significantly upregulated in control conditions are depicted in blue. B) As **A**, but illustrating the changes after 10 min of treatment. C) As **A**, but illustrating the changes after 30 min of treatment. D) As **A**, but illustrating the changes after 60 min of treatment. E) Histogram showing the relative abundance of PARP1 auto-modification. *n*=4 cell culture replicates, data is presented as mean values +/− SD.

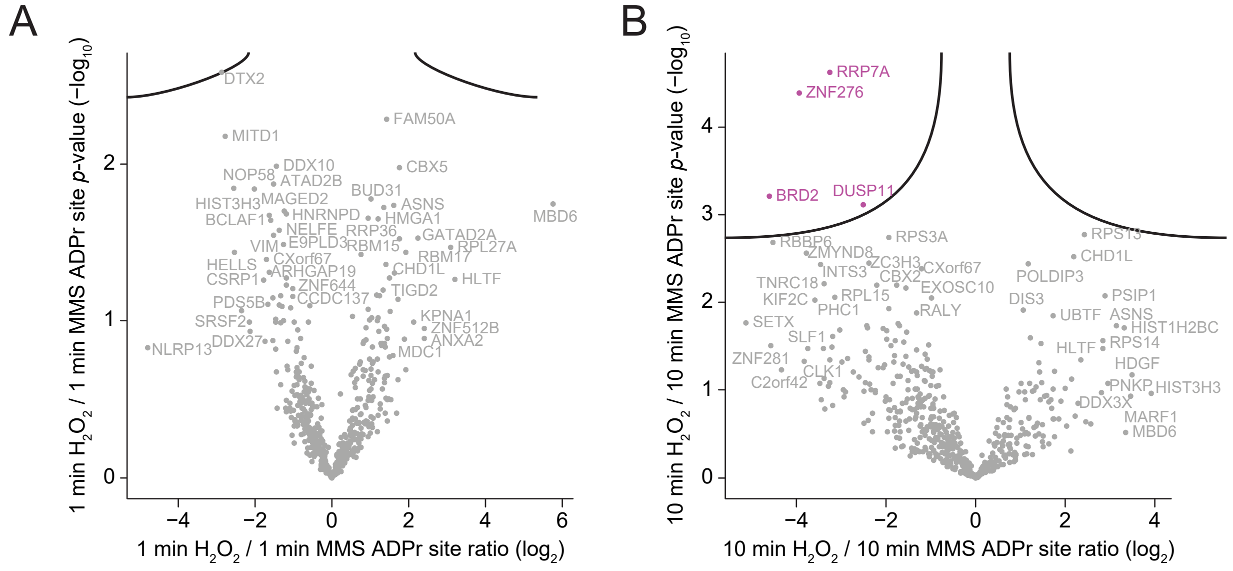
A
1 min H2O2 / 1 min MMS ADPr site p-value (−log10)
1 min H2O2 / 1 min MMS ADPr site ratio (log2)
DTX2
FAM50A
MITD1
DDX10
NOP58
ATAD2B
CBX5
BUD31
HIST3H3
MAGED2
ASNS
BCLAF1
HNRNPD
HMGA1
MBD6
NELFE
RRP36
GATAD2A
VIM
E9PLD3
RBM15
RPL27A
CXorf67
RBM17
HELLS
ARHGAP19
CHD1L
CSRP1
ZNF644
HLTF
PDS5B
CCDC137
TIGD2
SRSF2
KPNA1
DDX27
ZNF512B
NLRP13
ANXA2
MDC1
B
10 min H2O2 / 10 min MMS ADPr site p-value (−log10)
10 min H2O2 / 10 min MMS ADPr site ratio (log2)
RRP7A
ZNF276
BRD2
DUSP11
RBBP6
RPS3A
RPS13
ZMYND8
CHD1L
ZC3H3
CXorf67
INTS3
CBX2
POLDIP3
TNRC18
EXOSC10
KIF2C
RPL15
DIS3
PSIP1
PHC1
RALY
UBTF
ASNS
SETX
HIST1H2BC
SLF1
RPS14
HLTF
ZNF281
HDGF
CLK1
C2orf42
PNKP
HIST3H3
DDX3X
MARF1
MBD6

FIGURE S4

**Figure S4. Temporal-specific changes in the ADP-ribosylome.** A) Volcano plot analysis visualizing the dynamics of ADPr target proteins after 1 minute of $H_2O_2$ treatment compared to 1 minute of MMS treatment. Significance was determined via two-tailed Student's t-testing, with a FDR of 0.05, an s0 of 0.1, and 2,500 rounds of randomization. Proteins not significantly regulated are shown in grey. B) As **A**, but illustrating the changes after 10 minutes of treatment. Proteins significantly upregulated by MMS treatment are depicted in pink.

**REFERENCE**

Jungmichel, S., Rosenthal, F., Altmeyer, M., Lukas, J., Hottiger, M.O., and Nielsen, M.L. (2013). Proteome-wide identification of poly(ADP-Ribosyl)ation targets in different genotoxic stress responses. Mol Cell *52*, 272-285.
